# The effect of eccentric exercise-induced delayed onset neck muscle soreness on force steadiness and the spatial distribution of neck extensor muscle activity

**DOI:** 10.1038/s41598-026-49353-x

**Published:** 2026-04-30

**Authors:** Hirofumi Sageshima, Michail Arvanitidis, Georgios Sidiropoulos, Yaron River, Deborah Falla

**Affiliations:** 1 Department of Physiotherapy, Faculty of Physical Education and Sport, Charles University, Prague, Czech Republic; 2 Centre of Precision Rehabilitation for Spinal Pain (CPR Spine), School of Sport, Exercise and Rehabilitation Sciences, College of Life and Environmental Sciences, University of Birmingham, Birmingham, UK; 3 Health Assessment and Quality of Life Research Lab, Physiotherapy Department, School of Health Sciences, University of Thessaly, Lamia, Greece; 4 Hillel Yaffe Medical Centre, Technion School of Medicine, Hadera, Haifa, Israel

**Keywords:** Delayed onset muscle soreness, High-density surface electromyography, Spatial distribution of muscle activity, Entropy, Neck pain, Anatomy, Health care, Medical research, Physiology

## Abstract

This study investigated the effects of eccentric exercise-induced delayed onset muscle soreness (DOMS) on neck extensor muscle behaviour and force output, examining force steadiness and the spatial distribution of splenius capitis (SCap) and upper trapezius (UT) muscle activity. Twenty healthy individuals completed three laboratory sessions (baseline, 24 h and 48 h post-exercise), where participants performed submaximal isometric neck extension and flexion tasks (20%, 50%, and 70% of their maximum voluntary contraction). High-density surface electromyography was used to examine bilateral SCap and UT muscle activity, and force steadiness was evaluated using the absolute and relative amplitude of force fluctuations. Neck muscle soreness significantly increased 24 h and 48 h post-exercise compared with baseline (*p* < 0.001), along with significant reductions in pressure pain thresholds over suboccipital and C4 regions (*p* < 0.001). Caudal shifts of SCap and UT muscle activity were observed during neck extension contractions in the presence of DOMS (*p* < 0.01, *p* < 0.001, respectively), and entropy values were significantly higher at 24 h and 48 h for the SCap (*p* < 0.001). The absolute amplitude of force fluctuations during the neck extension contractions improved across sessions (*p* < 0.05). These findings suggest that acute neck muscle soreness induces a redistribution of neck muscle activity; this is likely to be adaptive to protect painful muscle regions.

## Introduction

Individuals with chronic neck pain (CNP) commonly exhibit a wide range of functional impairments compared with asymptomatic individuals, including reduced maximal range of neck motion^1^, reduced neck muscle strength^2^, impaired force steadiness^3–5^, poor proprioception^6^, reduced visuomotor reaction time^7^, and altered movement variability^1,8,9^. These impairments can contribute significantly to disability and reduced quality of life and may play a role in the recurrence or persistence of neck pain. Among these factors, force steadiness represents a key indicator of neuromuscular control^10^ and is defined as the ability to maintain a consistent level of force during a submaximal voluntary contraction. Precise and smooth control of force output is essential, not only for functional mobility, but for maintaining joint stability. Furthermore, inadequate modulation of muscle force can result in suboptimal tissue loading and increased mechanical stress on joints and passive structures, potentially contributing to persistence of pain^11^.

A recent meta-analysis demonstrated that force steadiness is commonly impaired in individuals with chronic musculoskeletal pain^12^, including those with CNP^4,5^. Furthermore, individuals with CNP commonly exhibit alterations in muscle coordination^13,14^, increased muscle coactivation^15^, and change in the spatial distribution of muscle activity^16,17^, all of which may influence the accurate control of force.

While investigating neuromuscular control in people with chronic pain provides valuable insight into long-term adaptations to pain, it remains unclear whether similar changes occur immediately following the onset of acute pain. Specifically, it is unknown whether reductions in force steadiness and alterations in muscle activation patterns represent rapid protective adjustments or if they develop gradually as pain becomes persistent. Understanding immediate adaptations to pain is essential for identifying mechanisms that may contribute to the transition from acute to chronic pain and for developing early and targeted exercise interventions. Experimental pain models provide an effective framework to investigate acute neuromuscular adjustments to pain under controlled conditions, allowing for the isolation of pain-specific effects on neuromuscular function e.g., without psychological features which commonly accompany acute clinical pain.

A recent systematic review which synthesised evidence on the effect of experimental pain models applied within the neck region on cervical neuromuscular control and neck kinematics, revealed that acute experimental pain can induce rapid alterations in muscle activation, such as a caudal shift in upper trapezius activity^18^. The majority of studies in this systematic review used hypertonic saline as the experimental pain model. Similarly, a meta-analysis on the effects of experimental pain on force steadiness^12^ identified only 13 studies, almost all of which employed hypertonic saline or ascorbic acid, and none of these studies investigated the effects of experimental pain on neck force control. These models evoke short-lasting, localised pain that differs substantially from the duration and quality of clinical musculoskeletal pain^19^.

To overcome these limitations, another approach is to use eccentric exercise to induce delayed onset muscle soreness (DOMS). DOMS is a transient form of exercise-induced muscle pain that develops within 24–72 h following unaccustomed eccentric contractions^20,21^. It is characterised by reduced force production, stiffness, swelling, and soreness during movement rather than at rest^22,23^. Given that the soreness/pain produced following eccentric exercise is longer-lasting and activity-related, it more closely mimics the characteristics of clinical musculoskeletal pain^24,25^.

Recent research has successfully applied eccentric exercise-induced DOMS to investigate trunk muscle control. For example, Arvanitidis et al. (2024) demonstrated that DOMS in the lumbar extensor muscles alters high-density surface EMG–torque relationships and trunk kinematics, while Abboud et al. (2021) and Houle et al. (2020) reported changes similar to those observed in chronic low back pain in the presence of DOMS^26–28^. These findings provide a strong rationale for applying a similar experimental pain model to the cervical region to investigate acute neuromuscular adaptations to pain. Very few studies have investigated the effects of DOMS of the neck muscles on neck function. One example is the study by Alsultan et al. (2020), which showed that eccentric exercise-induced DOMS in the neck extensors, reduced the variability of active neck movements^29^; a finding which has been observed in people with CNP.

The aim of this study was to investigate how eccentric exercise-induced DOMS of the neck extensor muscles affects their function in relation to muscle behaviour and force control. Specifically, we examined changes in force steadiness, the spatial distribution of high-density surface EMG (HDsEMG) amplitude of the splenius capitis (SCap) and upper trapezius (UT) muscles, and the amount of antagonist extensor muscle activity during neck flexion following DOMS induction. It was hypothesised that, in the presence of DOMS of the neck extensors, participants would exhibit reduced force steadiness and an altered spatial distribution of muscle activity in the SCap and UT muscles together with increase coactivation, findings which are commonly observed in people with CNP conditions. This would lend support to these being early adaptations following the onset of pain and would have relevance to the prescription of early exercise interventions following the onset of acute neck pain.

## Methods

### Study design and setting

This was a laboratory-based, repeated-measures experimental study utilising an exercise-induced DOMS model to examine acute neuromuscular responses. The study was approved by the Ethics Review Committee at the University of Birmingham, United Kingdom (approval number: ERN_2068-Apr2024) and adhered to the Declaration of Helsinki. Data collection was conducted from April 2024 to June 2024 at a laboratory within the Centre of Precision Rehabilitation for Spinal Pain, School of Sport, Exercise and Rehabilitation Sciences, University of Birmingham, United Kingdom. Each participant attended three sessions on consecutive days, approximately 24 h apart (baseline, 24 h and 48 h post-exercise) and all participants gave their written informed consent before participation. The reporting of EMG measures and results adheres to the CEDE-Check^30^; a checklist to assist researchers to thoroughly report their EMG methodologies.

### Participants

Twenty asymptomatic participants were recruited from the local community of Birmingham, including students and staff of the University of Birmingham, thorough social media announcements and distributed information leaflets. Inclusion criteria were (a) men and women between 18 and 60 years of age; (b) free of shoulder and neck pain; and (c) no past history of orthopaedic disorders affecting the shoulder or neck region. Exclusion criteria were previous cervical spine injury (e.g. fracture) or spinal surgery, neurological disorders, rheumatological conditions, pregnancy, or any cardiopulmonary diseases. Moreover, participants who performed any strenuous activity 24 h before the measurements were excluded from the study.

The sample size was estimated using G*Power 3.1 for Windows (Heinrich Heine University Düsseldorf, Germany; https://www.psychologie.hhu.de/arbeitsgruppen/allgemeine-psychologie-und-arbeitspsychologie/gpower), assuming a medium effect size (f = 0.33), an α of 0.05, a β power of 0.8, and a 10% data loss due to poor signal quality or participant withdrawal, for a two-way repeated measures analysis of variance (ANOVA), using session and force level as within subject factors. The effect size was derived from mean ± SD of the distribution of UT activity along y-axis between control and painful conditions following pain induced via injection of hypertonic saline^31^. This decision was based on the absence of studies quantifying the spatial distribution of SCap muscle activity under experimental pain conditions.

### Questionnaires

At the beginning of the first experimental session, participants were asked to complete questionnaires to assess their baseline characteristics in addition to collecting demographic data. Physical activity level was evaluated using the short form of the International Physical Activity Questionnaire (IPAQ), which estimates weekly MET (metabolic equivalent) values and categorizes levels of activity into low, moderate and high^32^. The reliability and validity of the short form of the IPAQ has been established^33^. The general health of the participants was assessed using the SF-36 (36-Item Short-Form Health Survey), which comprises eight physical and mental health domains and is scored from 0 (“severe disability”) to 100% (“no disability”). The reliability of this questionnaire for the measurement of physical and mental health has been established^34^.

Neck muscle soreness and current resting neck pain intensity were assessed at the beginning and end of the first session, as well as at the beginning of the second (after 24 h) and third (after 48 h) sessions. More specifically, participants were asked to actively move their neck (flexion, extension, lateral flexion, and rotation) and then rate the overall intensity of perceived neck extensor muscle soreness using a 0–100 mm visual analogue scale (VAS), where 0 represented “no soreness at all” and 100 represented “extreme soreness”^35^. Participants provided a global rating of muscle soreness reflecting general neck soreness during movement, not separately for the left and right sides. Additionally, they were asked to rate their level of resting neck pain intensity using a numerical pain rating scale (NPRS), ranging from 0 for no pain, and 10 for the worst possible pain imaginable^36^. Finally, participants were asked to report their perceived exertion at several time points throughout each experimental session using the Borg CR 10 scale, which ranges from 0 (nothing at all) to 10 (extremely strong)^37^. The reliability and validity of the Borg CR 10 scale for monitoring the perceived exertion during exercise has been well established^38^.

### Pressure pain thresholds

To assess changes in mechanical sensitivity associated with DOMS, pressure pain thresholds (PPTs) were measured at the beginning of each session, using an electronic algometer (NOD, OT Bioelettronica, Italy) with a 1cm^2^ rubber-tipped probe at an application rate of 30 kPa/s. Participants were instructed to indicate to the researcher when the sensation of pressure transitioned to pain, at which moment the pressure application ceased. All PPTs were performed on the right side at two predetermined sites with the participants in prone lying: (i) suboccipital muscle region, 2 cm lateral to the spinous process of C2; (ii) over the neck extensors at the level of C4, 1 cm lateral to the spinous process, as described previously^29^. Each site was tested twice in a random order, and the average values were taken forward for statistical analysis.

### Electromyography

**Fig. 1:**
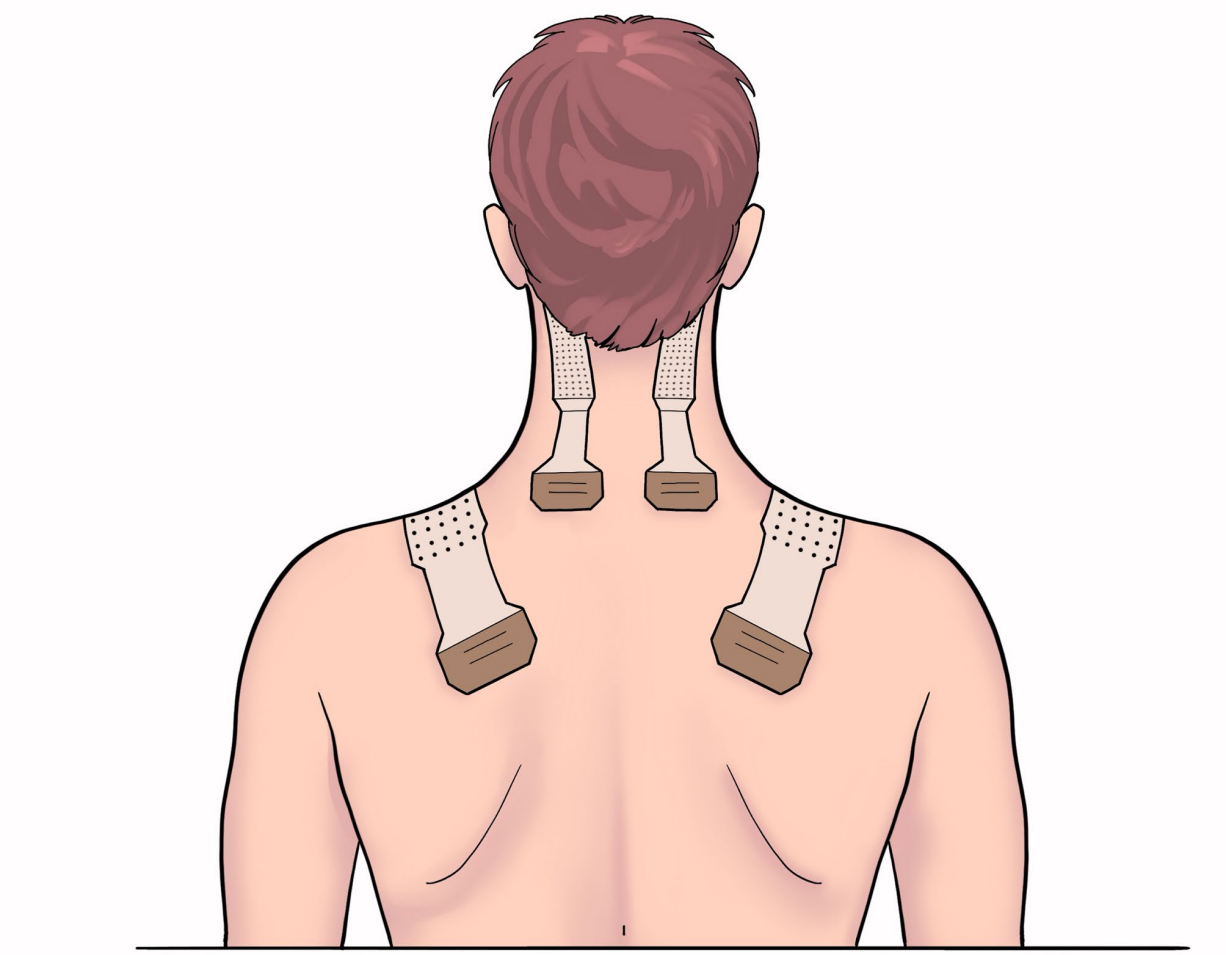
Location of HDsEMG electrode grids over the bilateral splenius capitis (SCap) and upper trapezius (UT). For the SCap, the grid was placed from C7 to C2 at the intersection between the SCM and UT along the muscle’s line of action. For the UT, the 4th row of the electrode grid was positioned in line with C7 and the acromion.

HDsEMG signals were recorded using four 13 × 5 semi-disposable adhesive electrode matrices. The electrode grids were placed over the SCap muscles (GR04MM1305, OT Bioelettronica, Torino, Italy; gold-coated electrodes, 1 mm diameter; inter-electrode distance: 4 mm), and UT muscles (GR08MM1305, OT Bioelettronica, Torino, Italy; gold-coated electrodes, 1 mm diameter; inter-electrode distance: 8 mm) bilaterally. All grids were prepared by attaching the appropriate double-sided adhesive foam (FOA04MM1305 for SCap grids and FOA08MM1305 for UT grids; OT Bioelettronica, Italy) and filling each electrode cavity with a highly conductive paste to ensure optimal skin–electrode contact (AC-CREAM, SPES Medica, Genoa, Italy). After skin preparation, which included shaving (if necessary), gentle local abrasion with abrasive paste (Spes Medica, Italy) to reduce skin impedance, and cleaning with water, the HDsEMG electrode grids were placed as follows: (1) for SCap, from C7 to C2 at the intersection between the sternocleidomastoid (SCM) and UT along the muscle’s line of action^39^; (2) for UT, the 4th row of the electrode grid was positioned in line with C7 and the acromion^40^. Reference electrodes (WhiteSensor WS, Ambu A/S, Ballerup, Denmark) were placed over the C7 spinous process and right acromion process, and a wrist strap (WS1, OT Bioelettronica, Torino, Italy) was also used as a reference electrode. The placement of HDsEMG electrode grids is shown in Fig. 1. The HDsEMG were synchronized with the force signals (see below), which were extracted via an Ethernet cable connected to a Forza device (OT Bioelettronica, Turin, Italy), which amplified the force signal (sampling rate: 100 Hz). Both force and HDsEMG signals were digitised using a 16-bit A/D converter (Quattrocento, 400-channel EMG amplifier, OT Bioelettronica, Torino, Italy, amplification: 150, frequency range 10–500 Hz, first order, 3 dB). Data acquisition was performed using the OTBiolab + software version 1.5.9 platform (OT Bioelettronica, Italy; https://otbioelettronica.it).

### Multi-Cervical Unit (MCU) dynamometer

The Multi-Cervical Unit (MCU; BTE Technologies, USA) was used to assess neck extension and flexion isometric maximum voluntary contraction (MVC), isometric submaximal force steadiness tasks, and was also used for the eccentric exercise task to induce DOMS. Measurements with the MCU have been shown to have high reliability and validity with ICCs for MVCs ranging from 0.95 to 0.99^41^.

Participants were seated and stabilised using straps across the chest to minimise upper-trunk compensation during force exertion. For neck flexion measurements, the force cell of the MCU device was positioned just above the participants’ eyebrows, with a stabilisation band placed behind the occipital protuberance, and a stabilisation clamp securing the back of the head. For neck extension, the force cell was positioned perpendicular to the occipital protuberance, and a stabilisation band was secured just superior to the eyebrows. The positioning and setup of the MCU followed a protocol described previously^42^.

### Testing protocol

Participants were familiarised with the device and performed a brief warm-up consisting of several submaximal isometric contractions in the movement direction that had been randomly assigned to be tested first (either neck flexion or neck extension). Following this warm-up, they completed two MVCs in that same direction, each lasting 5 s and separated by 2 min of rest. The same procedure was then repeated for the opposite movement direction. The highest MVC value for each direction was used to define the corresponding submaximal force levels. After a 5-minute rest, participants performed two sustained isometric neck contraction at 20% MVC (2-s ramp-up and ramp-down with a 20-s hold phase), 50% MVC (5-s ramp-up and ramp-down with a 15-s hold phase) and 70% MVC (7-s ramp-up and ramp-down with a 10-s hold phase) in the movement direction that had been randomly assigned as the first to be tested. The order of the submaximal contraction was randomised, and each contraction was separated by a 2-minute rest. Real-time visual feedback of the force output and the target %MVC was provided via a computer monitor positioned 1.5 m in front of the participants. During the submaximal contractions, the participants were instructed to follow the trapezoidal visual feedback provided on the screen, and once the target %MVC was reached, maintaining their force as steadily as possible for the full duration of the hold phase.

At the end of session one, after completing all maximal and submaximal contraction tasks, the participants performed eccentric exercise of their neck extensors against a resistance of 40% MVC following a previously described protocol^29^. Participants were passively positioned in 45° of neck extension, and the load corresponding to 40% MVC was converted into pounds (lbs) and set using the MCU’s built-in weight stack. They were then instructed to push their head against the brace of the MCU to return it to a neutral position (0°), i.e., perform an eccentric contraction of the neck extensors. They completed three sets of 15 repetitions of this exercise with 1 min of rest in between sets. Immediately after the eccentric exercise protocol, participants rated their perceived muscle soreness using the VAS and the intensity of any resting neck pain using the NPRS.

During the second (24 h after the baseline session) and third (48 h after) sessions, neck extensor muscle soreness and resting neck pain intensity were again assessed along with PPTs. They then performed the same maximal and submaximal neck flexion and extension contractions as described above as HDsEMG was acquired from the SCap and UT.

### Data analysis

For the SCap, the electrodes were aligned vertically along the muscle fibers and processed offline to obtain 59 bipolar derivations, while, for the UT, bipolar derivations were obtained in the horizontal direction, resulting in a matrix of 13 × 4 and a total of 51 bipolar signals^43^. The signals were filtered using a 2nd order bandpass 20–350 Hz Butterworth filter before root-mean-square (RMS) amplitude calculations took place. Then, the signals were visually inspected to remove any channels with low signal-to-noise ratio. Less than 15% of channels were discarded per electrode grid. The RMS values were calculated for each of the bipolar recordings and then topographical maps of EMG amplitude were determined for each muscle and the x- and y-axis coordinates of the centroid of the EMG amplitude map were determined^44,45^. This allowed us to examine changes in the spatial distribution of muscle activity in the presence of DOMS. Additionally, the RMS values of all bipolar channels were averaged for each muscle to form one value that represents the global measure of myoelectric activity. Moreover, values of signal complexity (modified entropy) were calculated for each muscle and for each task; lower entropy values indicate greater heterogeneity and less uniform recruitment across the muscle, whereas higher entropy values indicate a more homogeneous activity^46^. Additionally, RMS values of the neck extensor muscle (SCap) during the neck flexion tasks were determined to investigate changes in antagonist activation across sessions during all maximal and submaximal contraction levels, providing insight into any potential increase in coactivation of the neck extension due to the presence of DOMS.

The peak force during each of the two MVC trials was used to determine maximal neck flexion and extension strength. To evaluate force steadiness, the absolute and relative amplitude of the force fluctuations were calculated, determined by the standard deviation (SD) and the coefficient of variance (CoV) of force, respectively^47^. These values were determined for each repetition of each submaximal contraction tasks and then averaged to provide a single value for each force level. The time windows selected for analysis were approximately 18s for 20% MVC, 13s for 50% MVC, and 8s for 70% MVC, aiming to exclude the first and last seconds of contraction during which participants typically overestimated or underestimated the requested force level.

All HDsEMG and torque signals were analyzed offline, using a custom script on MATLAB 2023a (The MathWorks Inc., MA, USA; https://www.mathworks.com/).

### Statistical analysis

Statistical analyses were performed using R version 4.3.1 (R Core Team, Vienna, Austria; https://www.r-project.org/) and RStudio version 2023.06.0 + 421 (Posit Software, PBC, Boston, MA, USA; https://posit.co/). The Shapiro-Wilk test was used to assess the normal distribution of the data, and the Lavene test was used to evaluate the assumption of homogeneity of variance. Given that these conditions were satisfied, parametric tests were used. Data are presented as mean and SD.

A linear mixed model (LMM) analysis was performed using the R package “lme4”^48^. The LMM analysis was performed separately for each muscle and each side. The muscle was not included in the model as a factor because the aim of the study was not to compare muscles but rather to examine within-muscle adaptations over time, and therefore each muscle was analysed independently. The following model was used for “variable of interest ~ session * (1| subject)”. The session (baseline, 24 h and 48 h) was used as a within-subject factor. The 1| subject is the random effect, which characterizes the variation that is due to individual differences. Considering that this was a repeated measures design, the random effect was added to resolve this nonindependence in the dataset^49^. Similarly, for the HDsEMG and force steadiness variables, the following model was used for “variable of interest ~ session * force level * (1| subject)”. The force level (20%, 50%, and 70%) and session (baseline, 24 h and 48 h) were used as a within-subject factor. The 1| subject is the random effect, which characterizes the variation that is due to individual differences. The relationship between the outcome variables (pain/muscle soreness vs HDsEMG variables and force steadiness) was also examined using repeated measures correlation analysis through the R package “rmcorr”^50^.

The normality of residuals was assessed using Shapiro-Wilk test after running the model. Residual outliers were removed using Cook’s distance method (threshold was 4 times the SD) when the normality assumption was not met^49^. Post-hoc pairwise comparisons were conducted using Tukey correction and least-squares contrasts, as implemented in the R package “emmeans”. The post hoc tests were evaluated based on the interactions identified on the continuous dependent variables. Post hoc results are reported as mean estimates (M) and 95% confidence intervals (CI). Statistical significance for all statistical analysis was set at *p* < 0.05.

## Results

### Participants

Twenty participants (six females and 14 males, aged 27.15 ± 4.56 years) completed all three sessions. The IPAQ results showed that none of the participants fell into the low physical activity category, with the majority classified as highly active. SF-36 scores across all domains were high, as expected for an asymptomatic and young adult sample. The characteristics of the participants are detailed in Table 1.

**Table 1:** Demographic Characteristics and Questionnaire Scores.

Characteristics	Mean ± SD
Sex, % female	30% (6:14)
Age, yr	27.15 ± 4.56
Height, m	1.75 ± 0.10
Weight, kg	74.06 ± 18.86
BMI, kg/m2	23.95 ± 4.17
IPAQ total METs score	4097.65 ± 3024.10
IPAQ physical activity category	High: 13; Moderate: 7; Low: 0
SF-36 General Health	86.55 ± 10.06
SF-36 Physical Function	99.00 ± 2.62
SF-36 Emotional well-being	79.80 ± 13.39
SF-36 Pain	92.13 ± 8.52

*BMI*: body mass index; *IPAQ*: International Physical Activity Questionnaires; *METs*: Metabolic Equivalent of Tasks; *SF-36*: 36-Item Short Form Survey.

### Pain measurements

Participants reported mild neck muscle soreness when assessed both 24 h and 48 h following eccentric exercise compared to baseline (session effect: F = 22.44, *p* < 0.001, η^2^ = 0.56). Likewise, their level of resting neck pain intensity (NRS; 0–10) was higher when assessed at 24 h and 48 h post exercise compared to baseline (session effect: F = 8.70, *p* < 0.001, η^2^ = 0.32) as expected. However, no significant differences were observed between the 24 h and 48 h timepoints. Mean ± SD values of muscle soreness and resting neck pain intensity are reported in Table 2.

**Table 2:** Mean ± SD of muscle soreness, resting neck pain intensity scores and pressure pain thresholds (PPTs) assessed over the suboccipital and C4 regions across each session.

Soreness/Pain	Time point	Mean ± SD
Soreness (/100)	Baseline	0.00 ± 0.00
	24 h	26.25 ± 22.82 ***
	48 h	20.25 ± 16.34 ***
Pain (/10)	Baseline	0.00 ± 0.00
	24 h	1.40 ± 1.85 **
	48 h	1.30 ± 1.45 **

**PPTs**	**Time point**	**Mean ± SD (kPa)**
Suboccipital	Baseline	262.44 ± 78.07
	24 h	211.38 ± 76.34 ***
	48 h	221.35 ± 64.20 ***
C4	Baseline	290.19 ± 82.89
	24 h	213.33 ± 67.77 ***
	48 h	235.41 ± 65.74 ***

Data are presented at baseline, after 24 h and 48 h post-exercise. ***: *p* < 0.001 compared to the baseline values; **: *p* < 0.01 compared to the baseline values.

Pressure pain thresholds (Table 2) were significantly lower at 24 h and 48 h post eccentric exercise compared to baseline, for both the suboccipital and C4 regions (session effect: F = 14.52, *p* < 0.001, η^2^ = 0.43; F = 18.23, *p* < 0.001, η^2^ = 0.52, respectively). However, no significant differences in PPT values were observed between the 24 h and 48 h assessments. Collectively, these findings support the effectiveness of the eccentric exercise protocol in inducing DOMS.

### Maximal voluntary contraction

No changes were observed in maximum neck extension strength across sessions. Baseline values were 17.06 ± 5.88 lbs, with comparable values at 24 h (16.30 ± 5.83 lbs) and 48 h (16.27 ± 6.26 lbs) post-exercise (session effect: F = 0.46, *p* = 0.63, η² = 0.02). Similarly, no changes were observed in maximum neck flexion strength. Baseline strength was 10.60 ± 4.94 lbs, with comparable values at 24 h (10.44 ± 5.26 lbs) and 48 h (11.16 ± 4.54 lbs), and no significant session effect (F = 3.13, *p* = 0.06, η² = 0.16).

### Electromyography measures

#### Global EMG amplitude during neck extension/flexion contractions

**Fig. 2:**
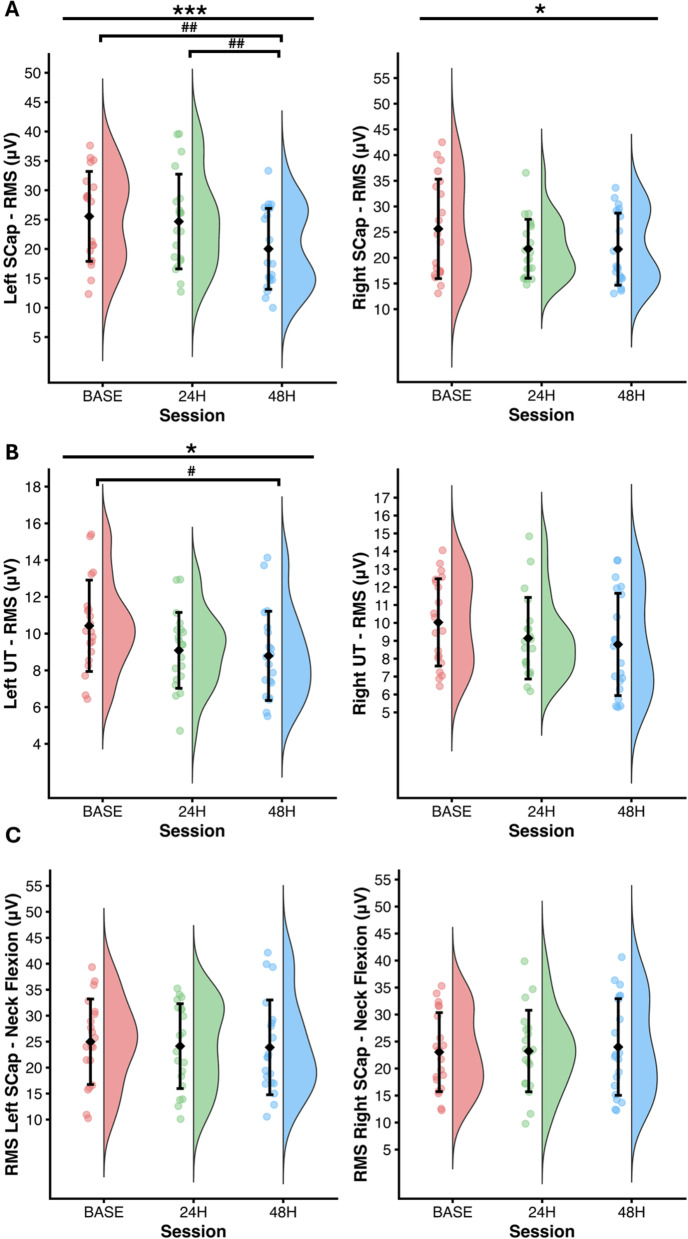
Average RMS values of left and right SCap (**A**) and UT (**B**) during neck extension and left and right SCap (**C**) during neck flexion at each force level and for each session. The RMS data has been pooled across force levels (20%, 50% and 70%). Red: Baseline; Green: 24 h post excentric exercise; Blue: 48 h post excentric exercise. Main effect of session, *: *p* < 0.05, ***: *p* < 0.001; post hoc pairwise comparisons with Tukey test, #: *p* < 0.05, ##: *p* < 0.01.

For the neck extension task, the global EMG amplitude of the SCap during the submaximal neck extension contractions differed across the three sessions for both the left and right side (session effect: F = 7.53, *p* < 0.001; F = 3.58, *p* = 0.03; Fig. 2A). Post-hoc analysis showed that left SCap activity progressively decreased across sessions, with lower activity observed at 48 h compared to both baseline and 24 h (baseline–48 h: MD = 5.53 (95%CI: 1.90;9.15); 24–48 h: MD = 4.65 (95%CI: 1.03;8.28), respectively; Fig. 2A). Right SCap activity did not differ significantly across sessions (baseline–24 h: MD = 3.88, 95% CI: −0.12;7.88; baseline–48 h: MD = 3.95, 95% CI: −0.05;7.96; Fig. 2A). No force level × session interaction was observed for either side (left: F = 0.61, *p* = 0.66; right: F = 0.77; *p* = 0.54).

Although no force level × session interaction was observed for the left or right UT (left: F = 0.22; *p* = 0.93; right: F = 0.82, *p* = 0.51), the level of left UT activity during the submaximal neck extension contractions was session-dependent (session effect: F = 4.44, *p* = 0.01; Fig. 2B), with a reduction of muscle activity observed at 48 h compared with baseline (baseline–48 h: MD = 1.64 (95%CI: 0.25;3.02); Fig. 2B). In contrast, the activity of the right UT remained consistent across sessions (session effect: F = 2.96, *p* = 0.05; Fig. 2B).

During the maximal neck flexion contractions, no significant differences were observed in SCap antagonistic activity across the sessions for either side (left: F = 0.01, *p* = 0.99; right: F = 0.30, *p* = 0.74). Similarly, SCap antagonistic activity during the submaximal neck flexion contraction tasks did not differ significantly across the sessions (left: F = 0.03, *p* = 0.97; right: F = 0.57, *p* = 0.57; Fig. 2C). No force level × session interactions were not observed for either side (left: F = 2.42, *p* = 0.05; right: F = 0.95, *p* = 0.44). These findings indicate that DOMS did not influence SCap antagonist activity during the neck flexion tasks at any contraction level.

### Y-axis coordinate of the centroid of the EMG amplitude map

**Fig. 3:**
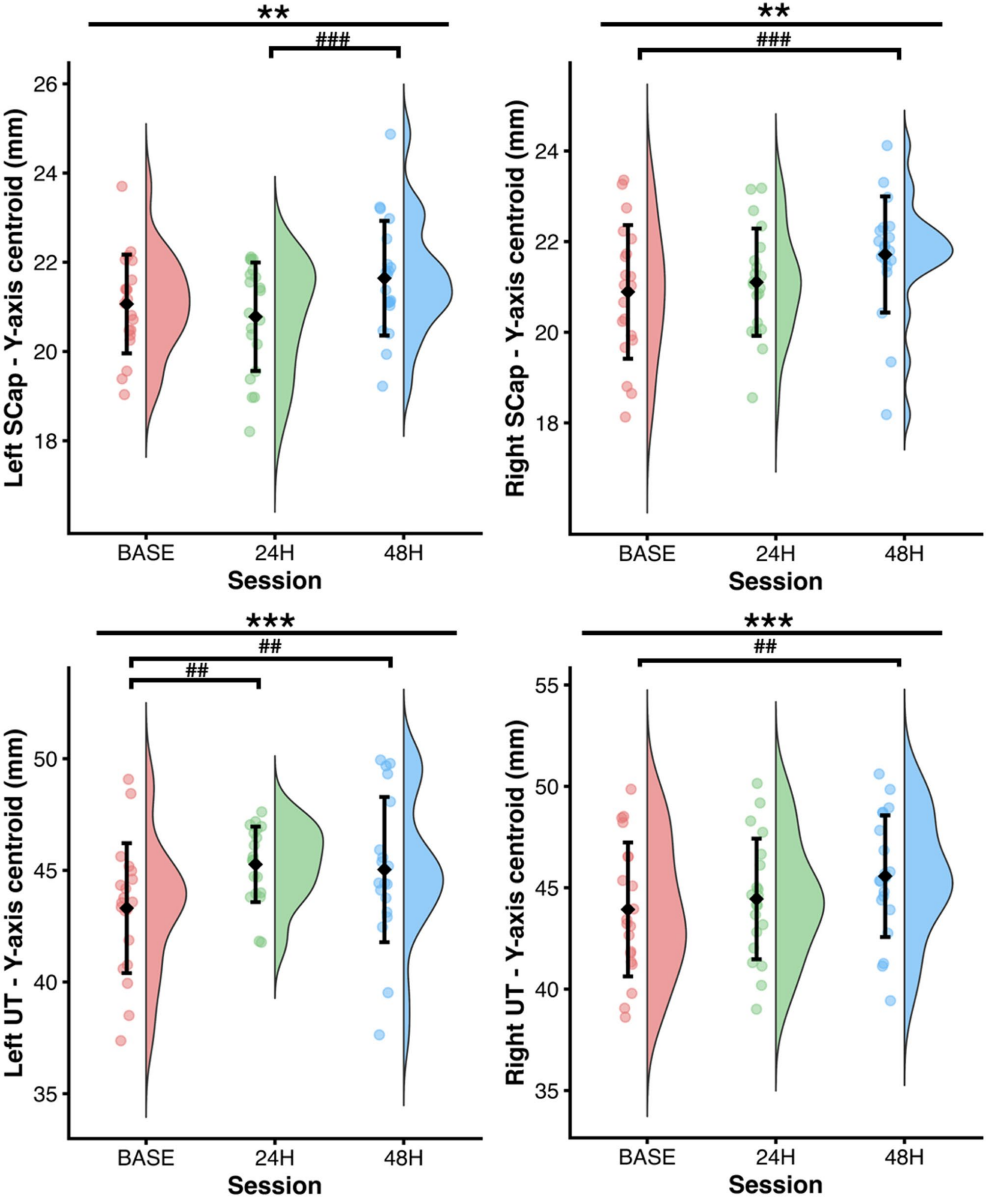
The y-axis coordinate of the centroid of left and right SCap and UT muscle activity during neck extension contractions. of each session and force level during neck extension contraction tasks. The y-axis coordinate data has been pooled across force levels (20%, 50% and 70%). Higer values mean the centroid of muscle activity shifts caudally. Red: Baseline; Green: 24 h post excentric exercise; Blue: 48 h post excentric exercise. Main effect of session, *: *p* < 0.05, **: *p* < 0.01, ***: *p* < 0.001; post hoc pairwise comparisons with Tukey test, #: *p* < 0.05, ##: *p* < 0.01, ###: *p* < 0.001.

The y-axis coordinate of the centroid of SCap activity differed significantly across sessions for both the left and right sides (session effect: F = 5.34, *p* < 0.01; F = 5.22, *p* < 0.01, respectively; Fig. 3). Post-hoc analysis indicated that the centroid of left SCap muscle activity was shifted more caudally at 48 h compared to 24 h, while for the right side, the centroid of muscle activity was shifted more caudally at 48 h compared to the baseline (left 24–48 h: MD = -0.86 (95%CI: -1.50;-0.23); right baseline–48 h: MD = -0.83 (95%CI: -1.45;-0.20); Fig. 3). However, no force level × session interaction was observed for either side (left: F = 0.72, *p* = 0.58; right: F = 0.79, *p* = 0.53).

For the UT, the y-axis coordinate of the centroid was also session-dependent, and this was the case for both sides (session effect: F = 7.79, *p* < 0.001; session effect: F = 4.06, *p* = 0.02, respectively; Fig. 3). For the left UT, a caudal shift of the centroid of muscle activity was observed at both 24 h and 48 h compared to the baseline (baseline–24 h: MD = -1.96 (95%CI: -3.24;-0.68); baseline–48 h: MD = -1.73 (95%CI: -3.01;-0.44); Fig. 3). Similarly, for the right UT, the centroid was positioned more caudally at 48 h compared to the baseline (baseline–48 h: MD = -1.64 (95%CI: -3.04;-0.25); Fig. 3). No force level × session interaction was found for either side (left: F = 1.12, *p* = 0.35; right: F = 1.04, *p* = 0.39). 

**Fig. 4:**
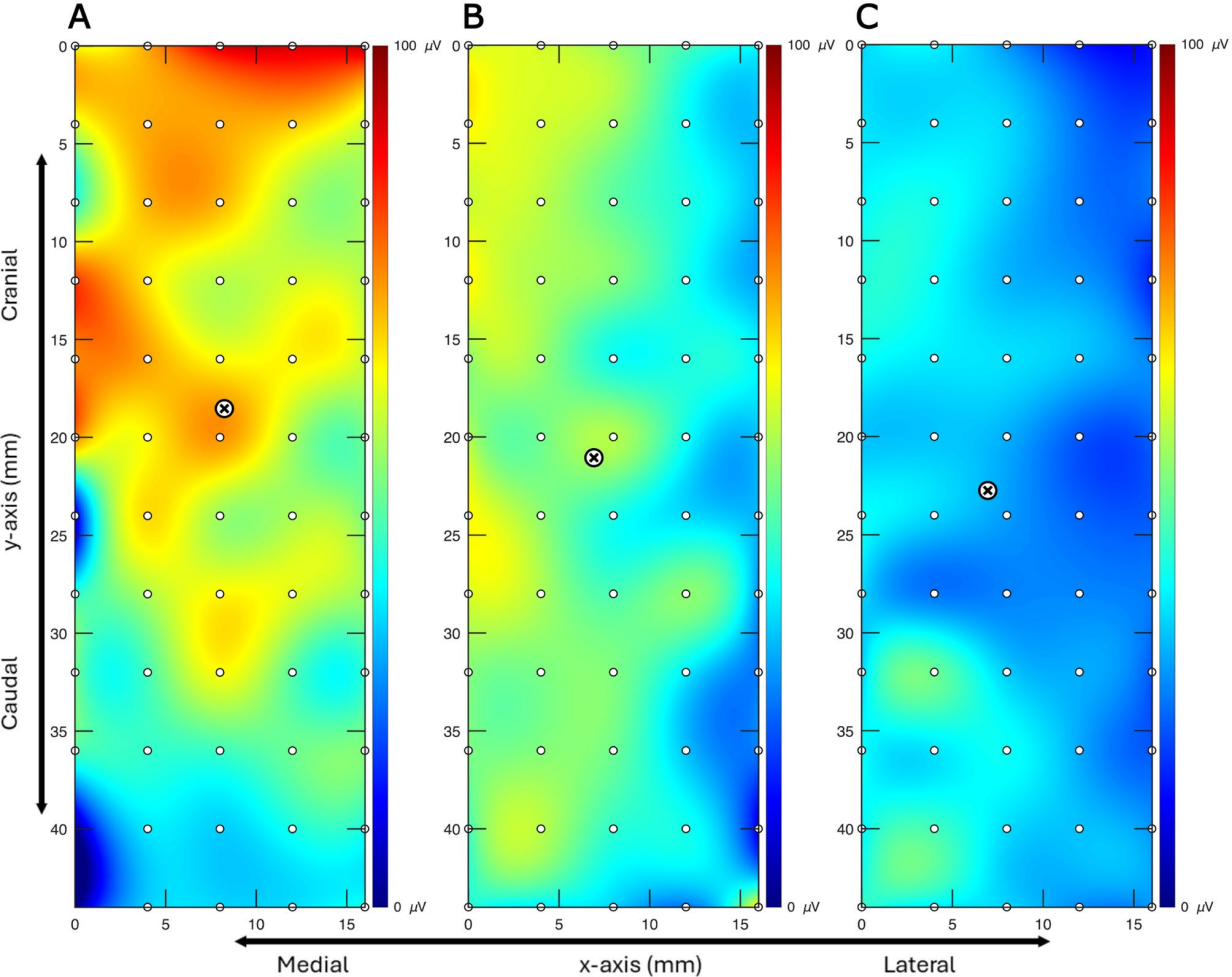
Representative example of the topographical maps of the right SCap at 70% MVC contraction task at the baseline (**A**), 24 h post-exercise (**B**), and 48 h post-exercise (**C**). It shows a caudal shift of the centroid of left SCap activity at 24 h. The centroid of muscle activity is depicted by a black cercle with “X”, and the scale is indicated in µV.

Figure 4 presents representative topographical maps of right SCap recorded at 70% MVC across the sessions. The topographical maps illustrate a caudal shift in the centroid of SCap activity observed at 24 h following the exercise, indicating an altered spatial distribution of SCap activity in the presence of the DOMS.

### X-axis coordinate of the centroid

**Fig. 5:**
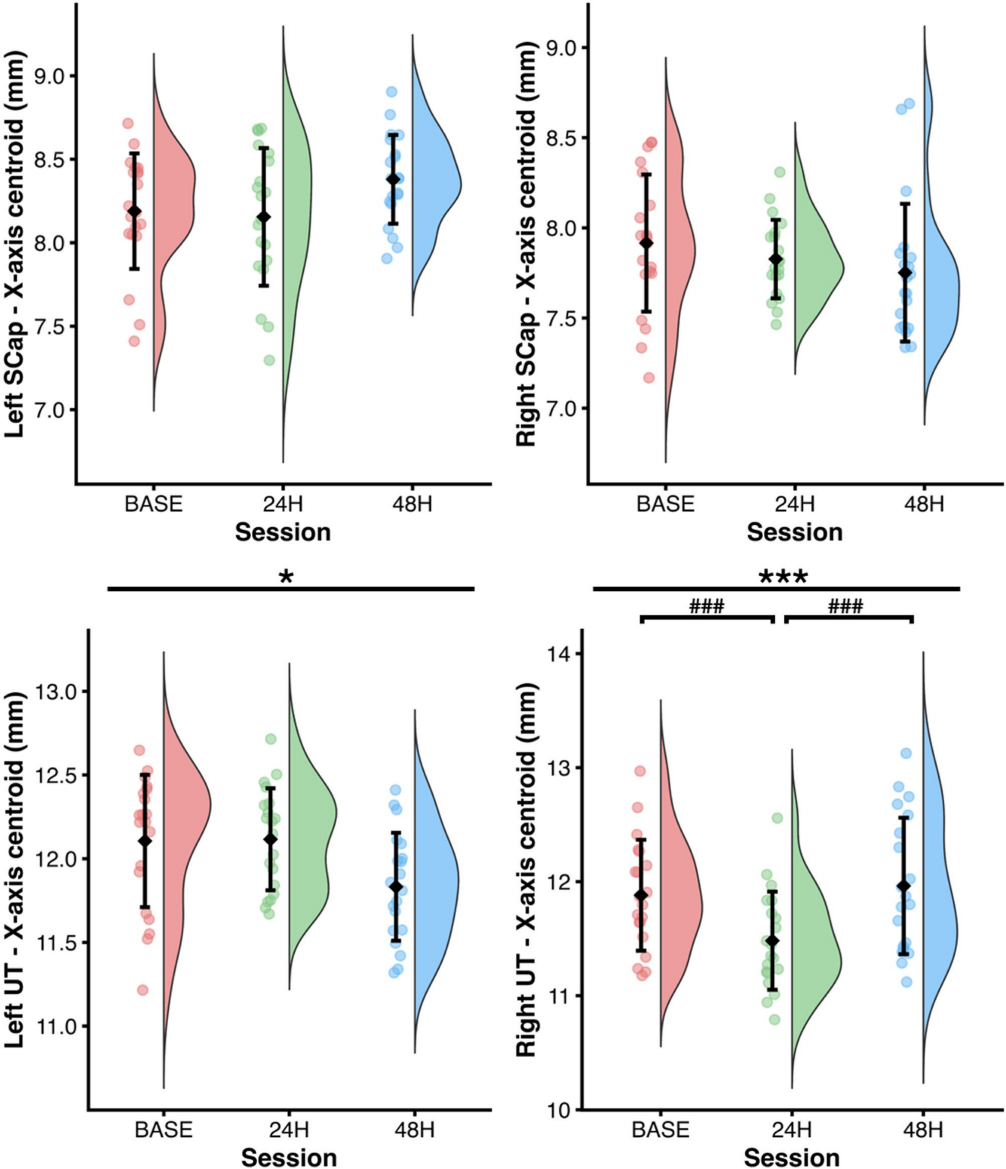
The x-axis coordinate of the centroid of left and right SCap and UT muscle activity of each session and force level during neck extension contraction tasks. The x-axis coordinate data has been pooled across force levels (20%, 50% and 70%). Higher values mean that the centroid of muscle activation shifts in the lateral direction for the left side of the muscles and shifts in the medial direction for the right side. Red: Baseline; Green: 24 h post excentric exercise; Blue: 48 h post excentric exercise. Main effect of session, *: *p* < 0.05, ***: *p* < 0.001; post hoc pairwise comparisons with Tukey test, ###: *p* < 0.001.

The x-axis coordinate values did not differ significantly across the three sessions for either left or right SCap (session effect left: F = 2.25, *p* = 0.11; right: F = 1.19, *p* = 0.31; Fig. 5). Likewise, no force level × session interaction was found for either side (left: F = 0.85, *p* = 0.50; right: F = 1.68, *p* = 0.16).

For the UT muscles, the x-coordinate values were session-dependent for both the left and right sides (session effect: F = 3.08, *p* = 0.048; F = 13.03, *p* < 0.0001, respectively). Post-hoc analysis indicated no significant differences for the left UT across sessions (baseline–48 h: MD = 0.27 (95%CI: -0.03;0.58); 24–48 h: MD = 0.28 (95%CI: -0.02;0.59); Fig. 5), but for the right UT, a significant lateral shift of the centroid was observed at 24 h compared with both baseline and 48 h (baseline–24 h: MD = 0.40 (95%CI: 0.16;0.64); 24–48 h: MD = -0.48 (95%CI: -0.72;-0.24); Fig. 5). No force level × session interaction was identified on either side (left: F = 1.35, *p* = 0.26; right: F = 0.58, *p* = 0.68).

### Entropy

**Fig. 6:**
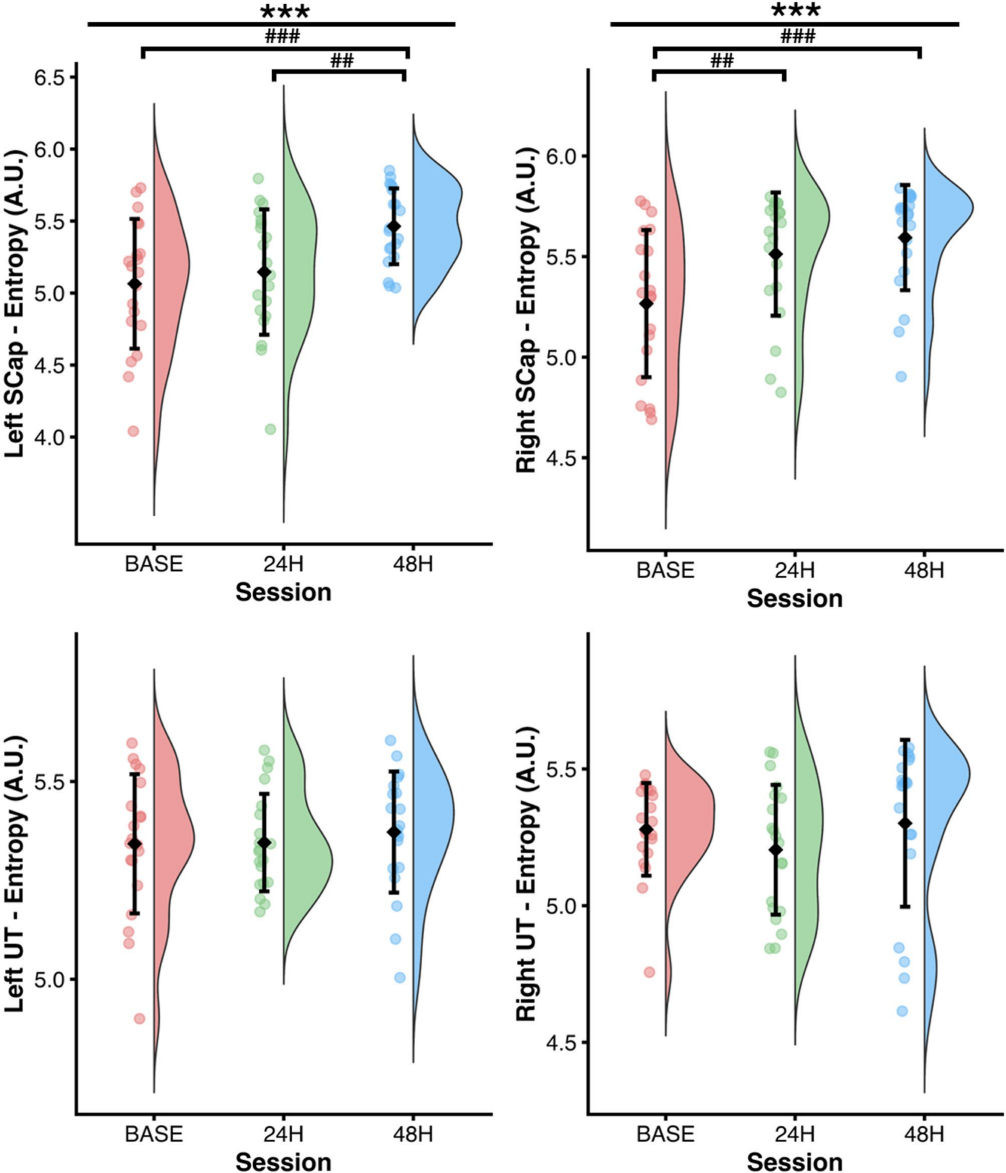
The entropy values of left and right SCap and UT of each session and force level during neck extension contraction tasks. The entropy data has been pooled across force level (20%, 50% and 70%). Higher values show more homogeneity of muscle activity. Red: Baseline; Green: 24 h post excentric exercise; Blue: 48 h post excentric exercise. Main effect of session, **: *p* < 0.01, ***: *p* < 0.001; post hoc pairwise comparisons with Tukey test, ##: *p* < 0.01, ###: *p* < 0.001.

The heterogeneity of muscle activity (modified entropy) differed significantly across sessions for both the left and right SCap (F = 10.62, *p* < 0.001, F = 10.56, *p* < 0.001, respectively). Post-hoc analysis showed that the left SCap entropy values were higher at 48 h than at baseline and 24 h (baseline–48 h: MD = -0.40 (95%CI: -0.62;-0.18); 24–48 h: MD = -0.32 (95%CI: -0.53;-0.10); Fig. 6), indicating greater homogeneity of muscle activity at 48 h. For the right SCap, the entropy values were significantly higher at 24 h and 48 h than at baseline (baseline–24 h: MD = -0.25 (95%CI: -0.42;-0.07); baseline–48 h: MD = -0.33 (95%CI: -0.50;-0.15); Fig. 6), indicating greater homogeneity of muscle activity in the presence of DOMS. No force level × session interaction was observed on either side (left: F = 0.25, *p* = 0.91; right: F = 0.36, *p* = 0.84).

For the UT, no significant differences in entropy were observed across the three sessions for either side (session effect left: F = 0.41, *p* = 0.67; right: F = 1.82, *p* = 0.17; Fig. 6). Similarly, no force level × session interaction was observed for either side (left: F = 0.31, *p* = 0.87; right: F = 2.05, *p* = 0.09).

### Force steadiness

**Fig. 7:**
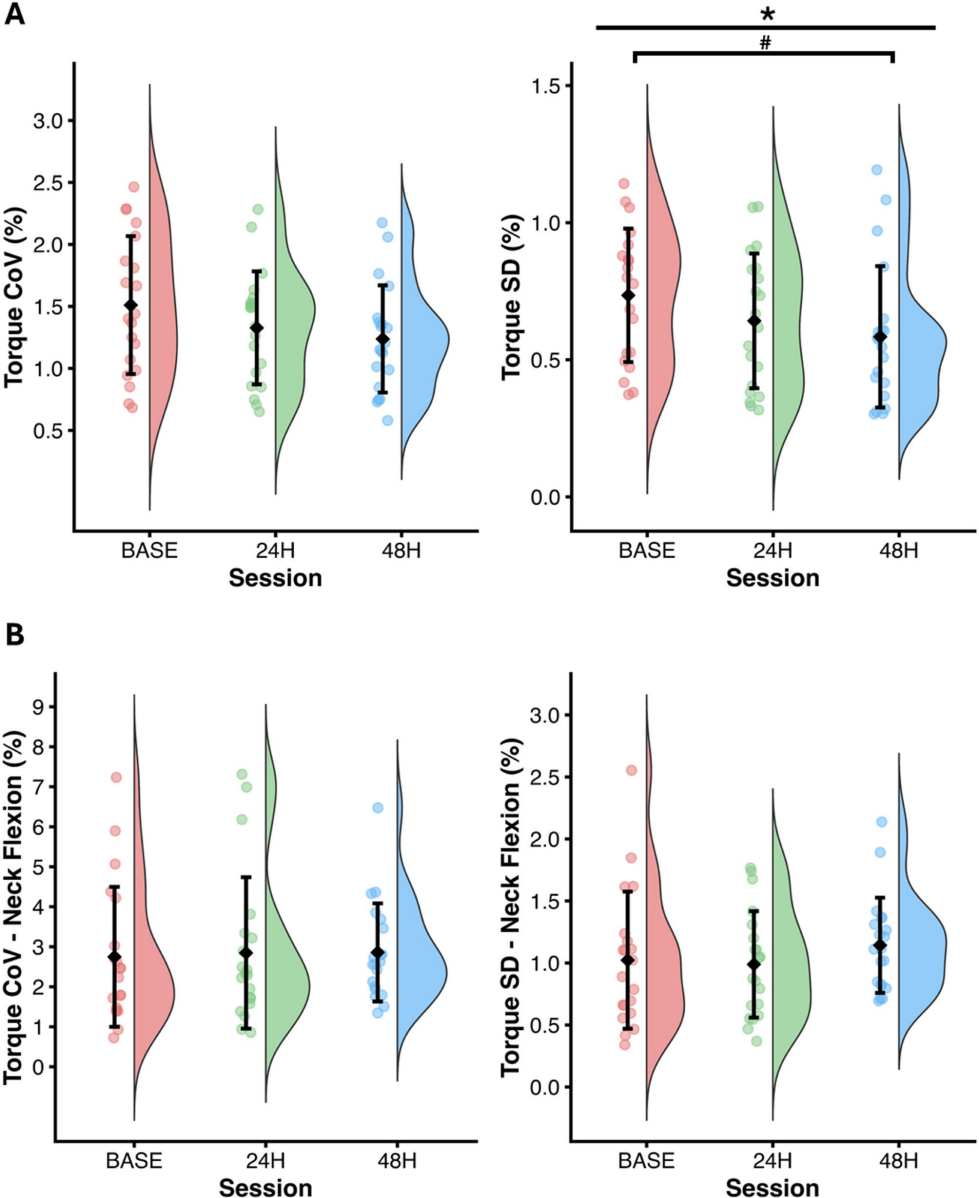
A: Torque CoV and torque SD of each session and force level during neck extension (**A**) and Flexion (**B**) contraction tasks. The torque CoV and SD data has been pooled across force levels (20%, 50% and 70%). Red: Baseline; Green: 24 h post excentric exercise; Blue: 48 h post excentric exercise. Main effect of session, *: *p* < 0.05; post hoc pairwise comparisons with Tukey test, #: *p* < 0.05.

For the neck extension tasks, no significant differences in the CoV of force were observed across sessions (F = 2.90, *p* = 0.06; Fig. 7A). In contrast, significant differences in the SD of force were observed, with baseline values being higher than those at 48 h (Session effect: F = 3.21, *p* = 0.04; Baseline–48 H: MD = 0.15, 95% CI: 0.01;0.29; Fig. 7A), suggesting an improvement in force steadiness performance at 48 h. No force level × session interaction was observed for either the CoV or the SD of force (F = 0.14, *p* = 0.97; F = 0.51, *p* = 0.73, respectively).

For the neck flexion tasks, no significant differences were found across sessions for either measure of force steadiness (CoV: F = 0.08, *p* = 0.92; SD: F = 1.21, *p* = 0.30; Fig. 7B). Likewise, no force level × session interaction was observed (CoV: F = 0.47, *p* = 0.76; SD: F = 0.73, *p* = 0.57).

### Association between pain and soreness with HDsEMG and force steadiness outcome variables

The association between neck pain/muscle soreness and HDsEMG outcome variables was examined. A weak but significant negative correlation was identified between muscle soreness and the global activity of the left UT (*r* = -0.19, 95%CI: -0.34;-0.04, *p* = 0.01), indicating that higher levels of muscle soreness were associated with lower UT HDsEMG amplitude. For the spatial distribution of muscle activity, a weak positive correlation was observed between the intensity of neck pain and the y-coordinate of the centroid for the left UT (*r* = 0.18, 95%CI: 0.02;0.32, *p* = 0.03), suggesting that greater levels of pain were associated with a more caudal shift in UT muscle activity on the left side. Additionally, muscle soreness showed a weak positive correlation with the entropy for the right SCap (*r* = 0.24, 95%CI: 0.09;0.38, *p* = 0.002), indicating that greater muscle soreness was accompanied by higher entropy values.

No significant correlations were identified between pain or muscle soreness and force steadiness for either the neck extension or neck flexion task.

## Discussion

This study evaluated the influence of DOMS on neck extensor force steadiness and muscle behaviour during submaximal isometric neck extension contractions. DOMS was successfully induced as demonstrated by the presence of resting neck pain and neck muscle soreness, and a decrease of PPT values when participants were assessed 24 and 48 h following the eccentric exercise protocol. In the presence of DOMS we observed: (i) lower activity of SCap bilaterally and UT on the left side, (ii) caudal shifts of the spatial distribution of SCap and UT bilaterally, (iii) and greater homogeneity of SCap activity. Additionally, there was an improvement in force steadiness during submaximal isometric extension contractions. However, no significant differences were observed in SCap activity during neck flexion at any force level.

The eccentric exercise protocol successfully induced DOMS, as evidenced by significantly increased muscle soreness and pain. These findings align with previous studies inducing DOMS in neck and shoulder muscles. The participants reported mild muscle soreness with average intensities of 26.25 and 20.25 on a 0–100 scale at 24 and 48 h, respectively, which is consistent with a previous study which induced DOMS of neck extensor muscles^29^. Additionally, the mean PPT values at both the suboccipital and C4 regions significantly decreased at 24 h and 48 h, which is also comparable to the study by Alsultan et al.^29^. The presence of muscle soreness/pain and mechanical sensitivity are most likely related to pathophysiological changes within the exercised muscle, including damage of subcellular structure of muscle fibres, including Z-band disruption, focal sarcomere damage, organelle displacement, and cytoskeletal disruption^51,52^. The microdamage of muscle fibres is followed by the release of inflammatory mediators, such as tumor necrosis factor, which leads to the sensitization of group III and IV afferent nerve endings^52,53^.

Maximal voluntary force of exercised muscles is commonly reduced immediately after eccentric exercise, and it can remain decreased when assessed 48 h later due to muscle soreness and fatigue^26,29,54^. However, no such reduction in the maximal neck extension or flexion force was observed when assessed 24 h and 48 h post-exercise. However, not all studies report a reduction in strength in the presence of DOMS; for example, a previous study reported no changes in the maximal force of the trunk extensors in the presence of DOMS^27^. The absence of strength decline is very likely explained by compensatory activation of other muscles, such as those within the upper thoracic region. Although we minimized potential compensations with verbal instructions and stabilization efforts using straps, it remained challenging to completely prevent participants from using compensatory movements during maximal efforts. Additionally, it is important to note that a learning effect may have contributed to the participants’ ability to maintain their MVC values across the three sessions^55^. For instance, repeated MVC testing over days without formal training has been shown to improve strength measures^56^.

An overall reduction of neck extensor muscle activity was observed in the presence of DOMS. While inconsistent findings have been reported for other muscles, such as the quadriceps^57^ and lumbar erector spinae^27^, no previous study has assessed neck extensor muscle activity in the presence of DOMS. Nevertheless, the DOMS-induced reduction in SCap activity is consistent with findings from previous studies examining the effects of experimental pain models, such as hypertonic saline injection into the UT, which resulted in reduced activity of painful neck muscles^40^, although inconsistent results are reported in the literature^18^. In the current study, it is likely that the reduced muscle activity is the result of reduced excitatory input to the population of motor neurons due to decreased descending drive to the muscle or/and to the spinal mechanisms triggered by nociceptive input^44^.

No significant differences were found in SCap activity during the neck flexion tasks, where this muscle functions as an antagonist. This was contrary to our hypothesis which was based on the observation of increased antagonist activity in individuals with CNP^15^. A previous study examining changes in agonist and antagonist neck muscle activity in response to hypertonic saline-induced neck pain showed that changes in the antagonistic muscle activity is task-dependent^40^. For instance, pain induced in the SCap resulted in reduced activity of SCap (agonist) and no changes in activity of the SCM (antagonist) during neck extension. Conversely, pain induced in the SCM led to decreased activity of SCM (agonist) and a decrease of SCap (antagonist) activity during neck flexion. This task-dependent nature of motor adaptations to pain could explain our results. Additionally, it should be noted that the pain experienced by the participants in the current study (pain intensity: 1.4 ± 1.9 at 24 h and 1.3 ± 1.5 at 48 h) was much less than the study which induced neck pain via hypertonic saline (peak pain intensity: 5.6 ± 1.9)^40^, which may also explain the lack of change in antagonist activity in the current study. It is therefore possible that the moderate eccentric loading protocol used in the present study (40% MVC) did not induce sufficient muscle soreness to provoke more substantial impairments in antagonist muscle activation. An eccentric exercise protocol involving higher resistance may have resulted in greater muscle damage and nociceptive input, potentially leading to more pronounced neuromuscular adaptations^2,58^.

The spatial distribution of neck extensor muscle activity differed in the presence of DOMS. Specifically, the centroid of activity of the neck extensors was shifted more caudally when DOMS was present, compared to the baseline condition. This change in the spatial distribution of activity was accompanied by increased entropy values when assessed 48 h post-exercise, indicating a more uniform activation pattern of the neck extensor muscles. The finding in this study aligns with several previous studies investigating the spatial distribution of UT activity with different pain experimental models. For instance, a caudal shift of UT activity has been observed following injection of hypertonic saline into the UT during sustained shoulder abduction tasks^17,31,59,60^ and a repeated lifting task^44^.

Further support for this observation comes from a previous study on individuals with myofascial pain and trigger points, where a more caudal shift in UT activity was reported compared to healthy controls during sustained shoulder elevation^16^. Similarly, people with fibromyalgia demonstrated a caudal shift of UT activity compared to pain-free participants during sustained shoulder abduction^17^. Such a caudal shift in UT activity may be explained by both motor unit recruitment characteristics and adaptive responses to nociceptive input. Motor units in the cranial region of the UT generally have higher recruitment thresholds, whereas those in the caudal region tend to have lower recruitment thresholds^44^. Therefore, motor units in the caudal region may be preferentially recruited when nociceptive input reduces the overall excitatory drive to the motoneuron pool. Additionally, from the perspective·of the protecting painful region, this redistribution of activity may help reduce mechanical stress in the painful or sensitized portion of the muscle. By shifting activation toward relatively less painful regions, the neuromuscular system may limit further stimulation of nociceptive afferents. Thus, this redistribution has been interpreted as an adaptive motor strategy aimed at protecting the painful region^61^.

We expected that DOMS would worsen force control, comparable to what is seen in people with CNP^3–5^. However, this was not the case and force steadiness improved 48 h after eccentric exercise, evidenced by significantly lower values in SD. Referring to results from other body regions, some studies reported no change in force steadiness in the presence of DOMS^27,62^. The improvement in force steadiness that we observed could be due to compensatory strategies such as using trunk extension when DOMS was present, which may have supported force production during the neck extension task. Additionally, a learning effect may have contributed to the observed improvement of the force accuracy task given that pain does not necessarily interfere with learning and adaptive processes^63,64^.

There are some limitations of this study which must be acknowledged. The sample consisted of young and physically active individuals, who may recover faster from eccentric exercise compared to older or less active individuals. Although mechanical hyperalgesia and muscle soreness confirmed the presence of DOMS, the intensity of muscle soreness and pain was relatively mild. Therefore, it is doubtful that these findings can be generalized to those experiencing more severe pain. Additionally, clinical neck pain, specifically chronic pain, involves a longer duration of symptoms, central adaptations, and·the presence of psychological features, which cannot be replicated by an acute experimental DOMS model^19^. Consequently, caution is warranted when extrapolating these findings to people with chronic neck pain conditions.

The eccentric exercise protocol applied in this study was based on previously published work^29^ but it may not have produced sufficiently high levels of DOMS to detect more pronounced neuromuscular alterations. A higher-resistance eccentric exercise protocol, or exercise performed until task failure, could have induced greater DOMS thereby inducing different neuromuscular adaptations. Additionally, only a single session of eccentric exercise protocol was applied in this study. Different levels of exposure·to eccentric exercise may have led to different magnitudes of DOMS, potentially also resulting in more pronounced pain-related alterations in muscle behavior. It is also important to note that the absence of a non-exercise control group prevents definitive conclusions regarding the observed neuromuscular adaptations to eccentric exercise-induced DOMS. Without comparison to a non-exercise group, it is difficult to fully exclude the contribution of potential confounders, such as learning or task familiarization effects, across sessions. Future studies should consider including multiple exposure intensities and a non-exercise control group to further understand the effects of DOMS on neuromuscular adaptations. As a further·consideration, this study did not measure potential compensatory patterns by kinematic analysis and recordings of synergic muscle activity. The lack of change in neck extension strength and force steadiness suggests that compensatory movements may have occurred.

Although chronic neck pain is more prevalent in females^65^, sex differences were not examined in the present study. The sample size was not powered to detect sex-related differences, and the unequal distribution of males and females limits meaningful subgroup analysis. Given that sex-related differences in pain sensitivity and motor control have been previously reported^66,67^, future studies should consider investigating whether neuromuscular responses to eccentric exercise-induced DOMS differ between sexes by using larger and more balanced samples. Furthermore, as muscle soreness was assessed as a global rating and not separately for the left and right sides, we could not evaluate whether laterality in perceived soreness was present or whether laterality-specific soreness may have contributed to the side-dependent differences observed in some HDsEMG outcomes. Future studies should assess the spatial distribution of soreness/pain to further explore associations between laterality-specific symptoms and neuromuscular adaptations. Finally, observers were not blinded during the data collection, which may have introduced bias and should be considered when interpreting the results.

The present findings indicate that people experiencing eccentric-exercise induced DOMS rapidly exhibit changes in the spatial distribution of SCap muscle activity, together with increased homogeneity of muscle activation. These findings suggest that acute muscle soreness or pain could lead to altered muscle activation strategies, likely reflecting a protective mechanism aimed at reducing the risk of further discomfort or sensitisation^61^. Such adaptations may be beneficial in the short term by reducing mechanical stress on sore muscle regions. However, our study did not directly test the functional consequences or long-term effects of these adaptations, and any interpretations regarding protective strategies or persistent changes remains unclear.

From a clinical perspective, these findings emphasise the importance of early identification of pain-related alterations in muscle activation and restoration of optimal neuromuscular function, before such changes resemble the maladaptive activation patterns commonly observed in people with CNP. For example, HDsEMG biofeedback could represent a useful approach to facilitate the restoration of muscle activation towards those observed prior to the onset of soreness, by promoting more optimal control and spatial organisations of muscle activity^68^. These strategies may help prevent the persistence of altered neuromuscular strategies and reduce the risk of acute neck pain progressing to chronic dysfunction and long-term impairment. Nevertheless, further studies are required to explore the clinical potential of these interventions.

## Conclusions

DOMS induces changes in the spatial distribution of neck extensor muscle activity with some observations consistent with what has been observed in people with CNP. These changes likely reflect an adaptive neuromuscular strategy to protect painful regions by redistributing load.

## Data Availability

Datasets generated and/or analyzed during the current study are available from the lead author (HS) upon reasonable request.
